# Targeted next-generation sequencing for pulmonary infection diagnosis in patients unsuitable for bronchoalveolar lavage

**DOI:** 10.3389/fmed.2023.1321515

**Published:** 2023-12-21

**Authors:** Zhenfeng Deng, Chunhong Li, Yingjin Wang, Fengwen Wu, Chunfang Liang, Wei Deng, Yuanli Wang

**Affiliations:** ^1^Clinical Genome Center, Guangxi KingMed Diagnostics, Nanning, China; ^2^Precision Medicine Laboratory, The First People’s Hospital of Qinzhou, The Tenth Affiliated Hospital of Guangxi Medical University, Qinzhou, China

**Keywords:** targeted next-generation sequencing, ultra-multiplex polymerase chain reaction, pathogenic diagnosis, pulmonary infection, conventional microbiological tests

## Abstract

**Background:**

Targeted next-generation sequencing (tNGS) has emerged as a rapid diagnostic technology for identifying a wide spectrum of pathogens responsible for pulmonary infections.

**Methods:**

Sputum samples were collected from patients unable or unwilling to undergo bronchoalveolar lavage. These samples underwent tNGS analysis to diagnose pulmonary infections. Retrospective analysis was performed on clinical data, and the clinical efficacy of tNGS was compared to conventional microbiological tests (CMTs).

**Results:**

This study included 209 pediatric and adult patients with confirmed pulmonary infections. tNGS detected 45 potential pathogens, whereas CMTs identified 23 pathogens. The overall microbial detection rate significantly differed between tNGS and CMTs (96.7% vs. 36.8%, *p* < 0.001). Among the 76 patients with concordant positive results from tNGS and CMTs, 86.8% (66/76) exhibited full or partial agreement. For highly pathogenic and rare/noncolonized microorganisms, tNGS, combined with comprehensive clinical review, directly guided pathogenic diagnosis and antibiotic treatment in 21 patients. This included infections caused by *Mycobacterium tuberculosis* complex, certain atypical pathogens, Aspergillus, and nontuberculous Mycobacteria. Among the enrolled population, 38.8% (81/209) of patients adjusted their treatment based on tNGS results. Furthermore, tNGS findings unveiled age-specific heterogeneity in pathogen distribution between children and adults.

**Conclusion:**

CMTs often fall short in meeting the diagnostic needs of pulmonary infections. This study highlights how tNGS of sputum samples from patients who cannot or will not undergo bronchoalveolar lavage yield valuable insights into potential pathogens, thereby enhancing the diagnosis of pulmonary infections in specific cases.

## 1 Introduction

Pulmonary infection stands as a prominent contributor to the global disease burden, resulting in substantial morbidity and mortality worldwide (1). Prompt identification of pathogens holds a crucial role in initiating timely and appropriate treatment, thereby enhancing clinical outcomes (2, 3). The prevailing diagnostic framework for pulmonary infection relies on physicians constructing a differential diagnosis from patient history, evaluating clinical presentations, interpreting imaging findings, conducting auxiliary tests, and subsequently employing microbiological assays to pinpoint causative agents. However, this conventional approach faces challenges in pathogenic diagnosis due to extended turnaround times, sensitivity, and the wide array of potential pathogens, encompassing bacteria, viruses, fungi, and atypical agents. Consequently, the etiology of pulmonary infections remains elusive in a substantial 19%–62% of cases (4–6).

Emerging as a technology with notable potential, targeted next-generation sequencing (tNGS) addresses the limitations of metagenomic NGS (mNGS) by targeting the identification of a broad spectrum of pathogens. Earlier investigations have underscored the efficacy of mNGS in significantly expediting pathogenic diagnosis in pulmonary infections (7, 8). Nevertheless, widespread clinical adoption of mNGS has been impeded by its considerable cost, susceptibility to host nucleic acid interference, and separate detection of DNA and RNA. In combination with ultra-multiplex polymerase chain reaction (PCR) and high-throughput sequencing, tNGS allows simultaneous identification of multiple common pathogens. Although the number of detectable pathogens is fewer than that detected using mNGS, tNGS offers distinct advantages in terms of the cost and diagnostic process. A preliminary report has demonstrated the effectiveness of tNGS in detecting respiratory pathogens, at a quarter of the cost of mNGS (9). Furthermore, another study revealed comparable diagnostic performance between tNGS and mNGS in microbiological testing of bronchoalveolar lavage fluid (BALF) (10).

Both BALF and sputum are commonly used sample types from the lower respiratory tract. Sputum, due to its noninvasive collection procedure and high patient acceptability, proves to be a more accessible option for early pathogenic screening than BALF. Therefore, sputum is often utilized for pathogen detection when obtaining BALF samples is infeasible or declined by patients. Consequently, tNGS of sputum holds promise as a pragmatic approach in such scenarios. However, the existing published evidence supporting the efficacy of sputum-based tNGS in patients with pulmonary infections is predominantly confined to small case series (11).

This study endeavors to elucidate the potential utility of tNGS in the pathogenic diagnosis of pulmonary infections. Employing a tNGS assay targeting 153 pathogens (Supplementary Table S1), we sought to assess its clinical performance in comparison to conventional microbiological tests (CMTs). Furthermore, we aimed to shed light on the heterogeneity of pathogen distribution within the study population based on tNGS results.

## 2 Materials and methods

### 2.1 Study design

This retrospective case series involved the analysis of 234 sputum samples, collected between April and November 2022 at The First People’s Hospital of Qinzhou in China. These samples were subjected to both tNGS and CMTs. Investigators conducted a thorough review of clinical data related to each patient diagnosed with pulmonary infection who underwent both tNGS and computed tomography scans. The study received approval from the local Ethics Committee (approval number: 2022081) and was conducted in accordance with the 1990 Declaration of Helsinki and its subsequent amendments. All data utilized in this study were obtained anonymously and exclusively employed for analysis in this paper. The confidentiality of patient information was rigorously upheld, obtaining the patient informed consent.

The inclusion criteria for this study were as follows: (i) patients diagnosed with pulmonary infection; (ii) implementation of both tNGS and CMTs for pathogenic diagnosis; (iii) availability of complete clinical data; and (iv) patients who provided informed consent to participate. Exclusion criteria included: (i) patients declining sample collection for tNGS; (ii) sputum samples failing to meet the tNGS quality standards; and (iii) patients with incomplete clinical data.

### 2.2 Sample collection

Adequate and informative sputum sample collection hinges on proper instructions. Trained nurses guided patients to brush their teeth and rinse their mouth with saline in the morning, take a deep breath, and then forcefully expel sputum from the respiratory tract, making an effort to avoid contamination with oral and nasopharyngeal secretions. The samples were collected in sterile containers with secure lids. Patients were explicitly instructed that sputum from a forceful cough was required, and saliva should not be introduced into the collection cup. For infants or young children unable to produce sputum through coughing, a disposable suction tube was employed to extract sputum under negative pressure. In cases where patients faced challenges in generating sputum through forced expectoration, alternative methods such as sputum induction and tracheal aspirate were considered. It is crucial to emphasize that all these procedures were exclusively conducted by physicians trained in accordance with specific collection protocols. Approximately 1–3 mL of sputum was collected and preserved at −20°C within 48 h for tNGS analysis. Additionally, residual sputum and blood samples from select patients were obtained for CMTs, including smear microscopy, culture, PCR, and serologic testing (Supplementary Presentation S1).

### 2.3 Targeted next-generation sequencing

#### 2.3.1 Sample preparation

A volume of 650 μL of the sample was liquefied by combining it with an equal volume of 80 mmol/L dithiothreitol in a 1.5 mL centrifuge tube. The mixture was homogenized for 15 s using a vortex mixer. Meanwhile, a positive control and a negative control from the Respiratory Pathogen Detection Kit (KS608-100HXD96, KingCreate, Guangzhou, China) were set up to monitor the whole experiment process of tNGS.

#### 2.3.2 Nucleic acid extraction

Subsequently, 500 μL of the homogenate was utilized for total nucleic acid extraction and purification via the MagPure Pathogen DNA/RNA Kit (R6672-01B, Magen, Guangzhou, China), following the manufacturer’s protocol.

#### 2.3.3 Library construction and sequencing

The library was constructed using the Respiratory Pathogen Detection Kit, and a no template control was set up to monitor the library construction and sequencing process. This process encompassed two rounds of PCR amplification. The sample nucleic acid and cDNA were employed as templates, and a set of 153 microorganism-specific primers were selected for ultra-multiplex PCR amplification to enrich the target pathogen sequences, spanning bacteria, viruses, fungi, mycoplasma, and chlamydia. After the amplification, PCR products underwent purification with beads, followed by amplification using primers containing sequencing adapters and distinct barcodes. The quality and quantity of the constructed library were evaluated using the Qsep100 Bio-Fragment Analyzer (Bioptic, Taiwan, China) and Qubit 4.0 fluorometer (Thermo Scientific, Massachusetts, United States), respectively. Generally, the library fragments exhibited sizes within the approximate range of 250–350 bp, and the library concentration was maintained at a minimum of 0.5 ng/μL. The concentration of the mixed library was reassessed and subsequently diluted to a final concentration of 1 nmol/L. Subsequently, 5 μL of the mixed library was mixed with 5 μL of freshly prepared NaOH (0.1 mol/L). Following brief vortexing and centrifugation, the library was incubated at room temperature for 5 min. The diluted and denatured library was subsequently subjected to sequencing on an Illumina MiniSeq platform using a universal sequencing reagent kit (KS107-CXR, KingCreate, Guangzhou, China). On average, each library yielded approximately 0.1 million reads, with a sequencing read length of single-end 100 bp.

#### 2.3.4 Bioinformatics analysis

Sequencing data were analyzed using the data management and analysis system (v3.7.2, KingCreate). The raw data underwent initial identification via the adapter. Reads with single-end lengths exceeding 50bp were retained, followed by low-quality filtering to retain reads with Q30>75%, ensuring high-quality data. The single-ended aligned reads were then compared using the Self-Building clinical pathogen database to determine the read count of specific amplification targets in each sample. The reference sequences used for read mapping was a database curated from different sources, including Genbank database, Refseq database, and Nucleotide database from NCBI.^1^

### 2.4 Interpretation of tNGS results

In line with the experimental principle of targeted amplification of microbial sequences using specific primers, the amplicon coverage and normalized read count of detected microorganisms within the sample constituted the primary interpretation indicators. To categorize a microorganism as a potential pathogen, the following criteria were established: (i) bacteria (excluding *Mycobacterium tuberculosis complex*), fungi and atypical pathogen: amplicon coverage ≥50% and normalized read count ≥10; (ii) viruses: amplicon coverage ≥50% and normalized read count ≥3, or normalized read count ≥10; (iii) *Mycobacterium tuberculosis complex*: normalized read count ≥1.

Subsequently, two experienced clinicians independently conducted a comprehensive assessment of the patient’s clinical data to determine the presence of pulmonary infection and the clinical relevance of potential pathogens. This assessment included the patient’s medical history, symptoms, imaging findings, tNGS results, and CMT outcomes. In cases of divergent interpretations, consultation with a senior physician was pursued to achieve a consensus.

### 2.5 Statistical analyses

Quantitative variables were represented as medians with accompanying ranges, while categorical variables were presented as counts with percentages. Statistical analyses were performed using SPSS 22.0 software (IBM, Armonk, NY, United States). A significance level of *p* < 0.05 was considered statistically significant.

## 3 Results

### 3.1 Patient characteristics

**Figure 1:**
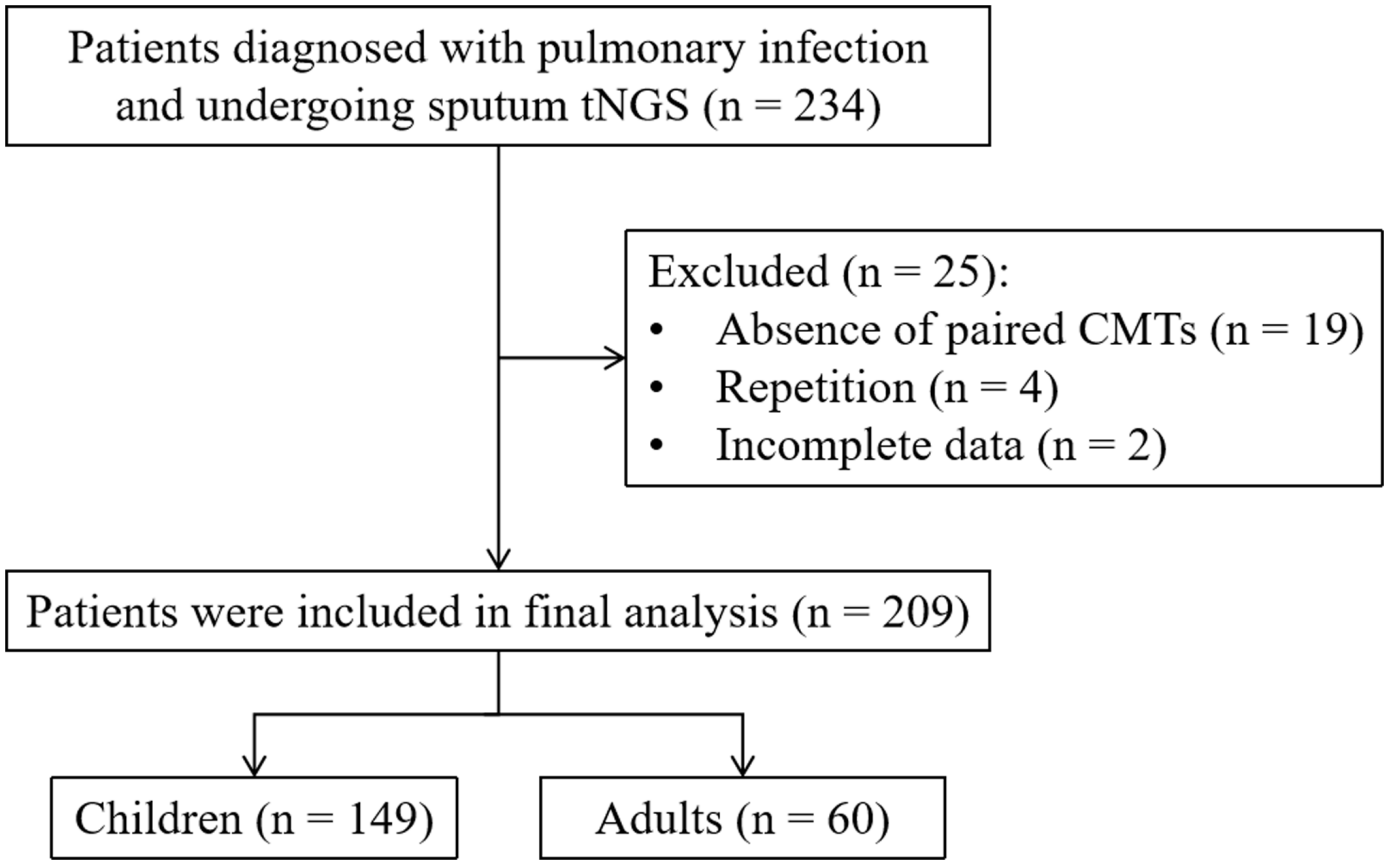
Flow diagram of the study.

Initially, a total of 234 patients diagnosed with pulmonary infection and under-going tNGS were considered for review and potential enrollment in this study. Among them, 25 patients were subsequently excluded due to reasons including the absence of paired CMTs (*n* = 19), repetition (*n* = 4), and incomplete data (*n* = 2). Consequently, a definitive cohort of 209 patients met the stipulated enrollment criteria and underwent further analysis (Figure 1). The median age of this cohort was 4 years, with 141 (67.5%) of the patients being male. Comorbidity was identified in 61.7% of these patients. A majority of patients (180, 86.12%) had been exposed to antibiotics before sample col-lection (Table 1).

**Table 1:** Baseline characteristics of the 209 patients enrolled.

Characteristic	Value (*n* = 209)
Median age, years	4 (0–97)
Distribution, *n* (%)	
< 18	149 (71.3)
≥ 18	60 (28.7)
Gender, *n* (%)	
Male	141 (67.5)
Female	68 (32.5)
Comorbidity, *n* (%)	
Yes	129 (61.7)
No	80 (38.3)
Antibiotic exposure, *n* (%)	
Yes	180 (86.1)
No	29 (13.9)

### 3.2 Pathogen detection using tNGS and CMTs

**Figure 2:**
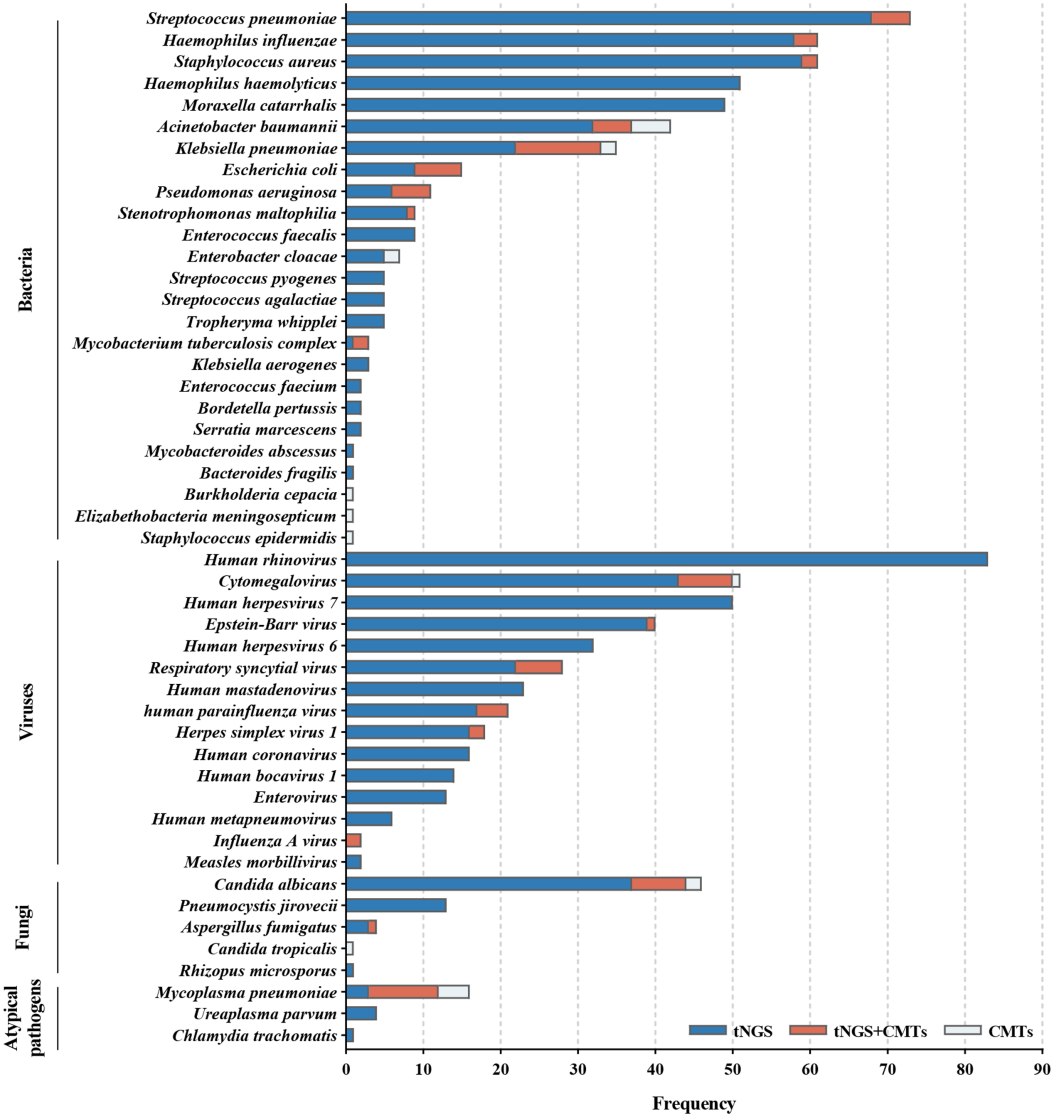
Distribution of potential pathogens in the study cohort and the respective contributions of tNGS and CMTs for pathogen detection.

**Figure 3:**
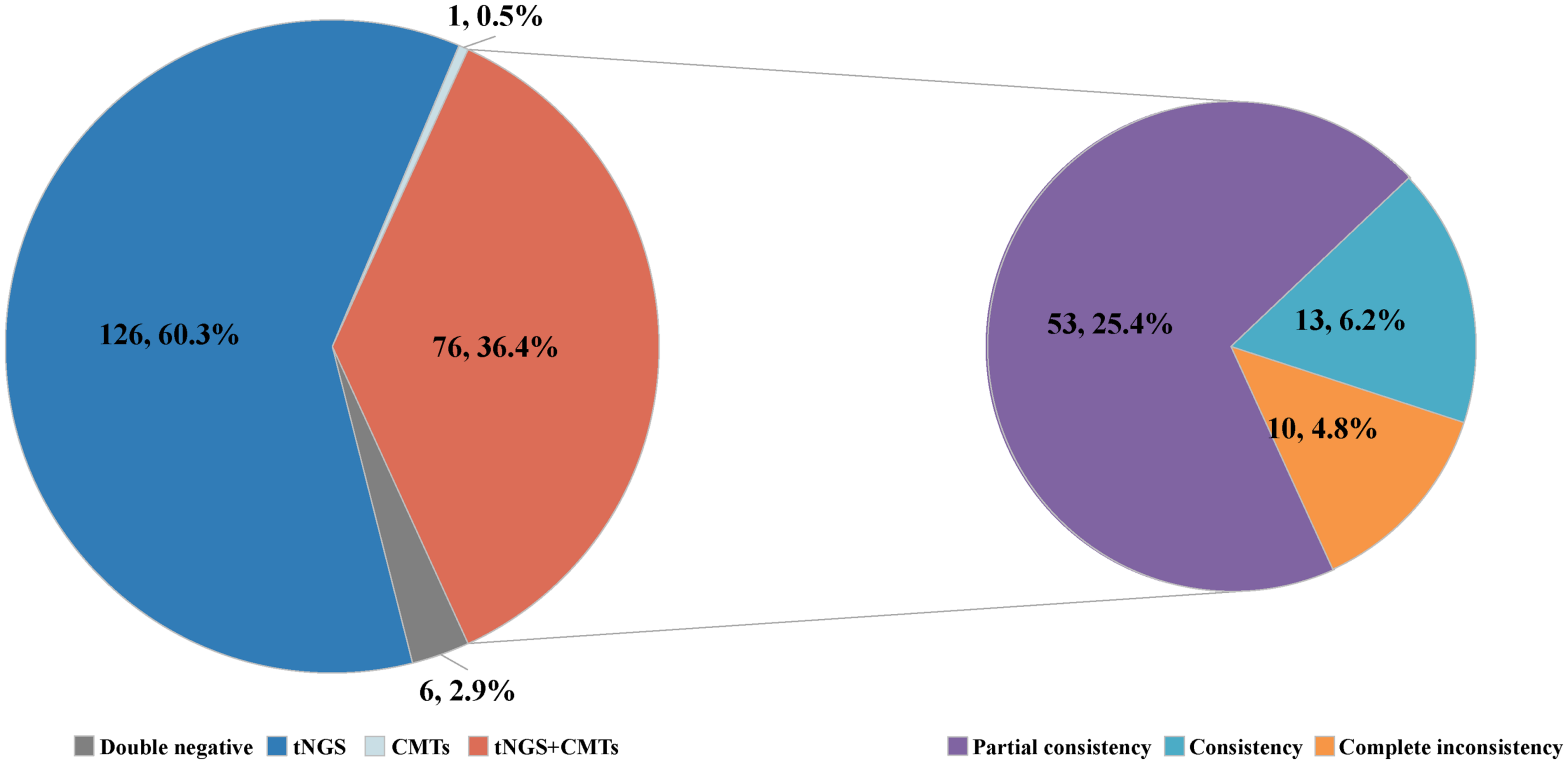
Consistency of pathogen detection results between tNGS and CMTs.

Among the 209 enrolled cases, a total of 45 potential pathogens were detected through tNGS, whereas CMTs identified 23 pathogens (Figure 2; Supplementary Table S2). The overall microbial detection rates for tNGS and CMTs were 96.7% (202/209) and 36.8% (77/209), respectively. Significantly, the detection rate of tNGS surpassed that of CMTs (*p* < 0.001). Of the total patients, 76 (36.4%) demonstrated positive results for both tNGS and CMTs, whereas 6 patients (2.9%) returned negative results in both methods. Additionally, 126 patients (60.3%) tested positive solely via tNGS, and 1 patient (0.4%) was positive exclusively via CMTs. Among the 76 double-positive patients, 13 patients (6.2%) displayed complete consistency between tNGS and CMT results, whereas 53 patients (25.4%) exhibited partial consistency, and 10 patients (4.8%) showed complete inconsistency (Figure 3).

Bacteria were identified in 184 patients (88.0%) via tNGS, whereas CMTs detected bacteria in 44 patients (21.1%). *Streptococcus pneumoniae* was the most frequently detected bacterium, accounting for 34.9% of total positive detections, followed by *Haemophilus influenzae* (29.2%), *Staphylococcus aureus* (29.2%), *H. haemolyticus* (24.4%), and *Moraxella catarrhalis* (23.4%).

Viruses were detected in 176 patients (84.2%) by tNGS and in 21 patients (10.1%) by CMTs. The predominant virus detected was human rhinovirus (HRV, 39.7%), followed by cytomegalovirus (CMV, 24.4%), human herpesvirus 7 (HHV-7, 23.9%), Epstein–Barr virus (EBV, 19.1%), and HHV-6 (15.3%).

Fungal detection was observed in 56 patients (26.8%) through tNGS and in 10 patients (4.8%) through CMTs. *Candida albicans* constituted 22.0% of positive detections, followed by *Pneumocystis jirovecii* (6.2%), *Aspergillus fumigatus* (1.9%), *Candida tropicalis* (0.5%), and *Rhizopus microsporus* (0.5%).

Atypical pathogens were identified in 16 patients (7.7%) by tNGS and in 13 patients (6.2%) by CMTs. This category included *Mycoplasma pneumoniae* (7.7%), *Ureaplasma parvum* (1.9%), and *Chlamydia trachomatis* (0.5%). Notably, among the 16 patients with confirmed *M. pneumoniae* infection, 12 exhibited positive tNGS results, and 13 exhibited positive serum IgM, with 9 patients demonstrating an overlap between the two.

Additionally, measles virus was detected through tNGS in 2 patients who had been vaccinated within 1 month; however, measles was not considered as a diagnostic consideration. Four microorganisms were solely identified via CMTs: *Elizabethella meningosepticum*, *Burkholderia cepacian*, *S. epidermidis*, and *C. tropicalis*. Each of these microorganisms was detected in only 1 patient within the study population, with the latter two not falling within the detection range of tNGS.

### 3.3 Heterogeneity of pathogen spectrum between children and adults

**Figure 4:**
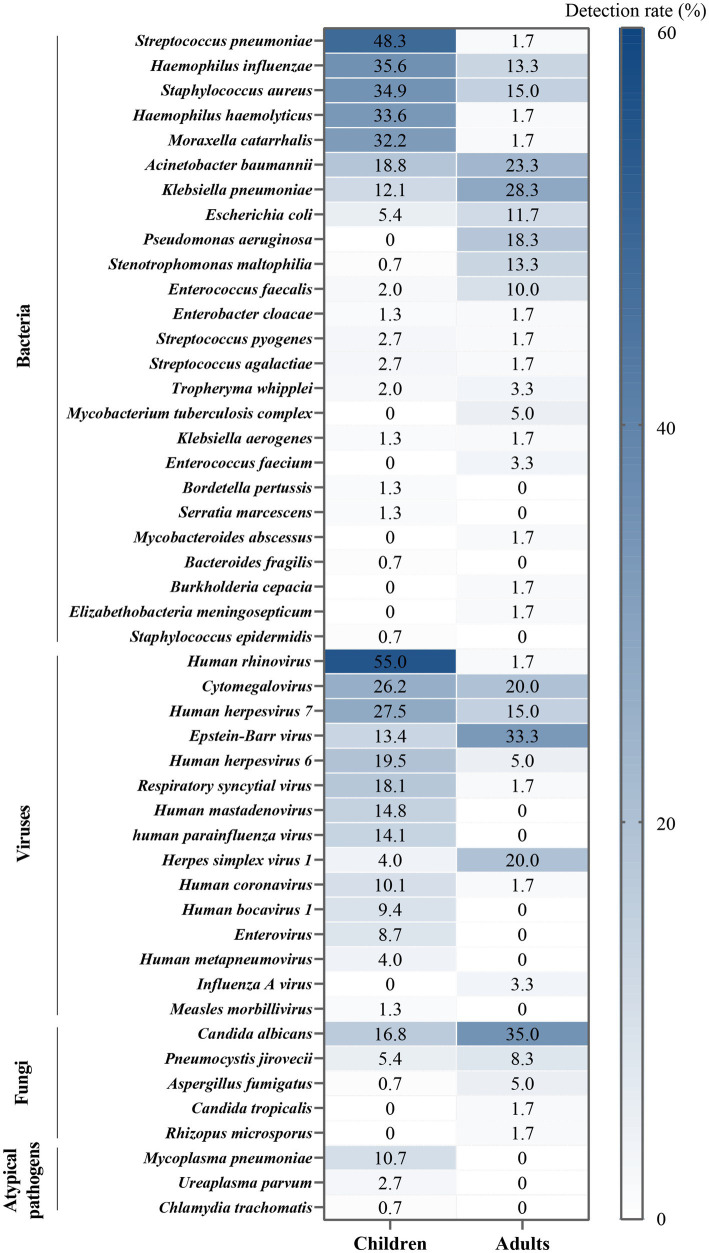
Heterogeneity in the pathogen spectrum between children and adults.

Within this study, notable heterogeneity was observed in the sputum pathogen spectrum when comparing children (age < 18 years) and adults (age ≥ 18 years) (Figure 4). Based on the proportion of positive detections, the predominant bacteria species among children were *S. pneumoniae, H. influenzae*, *S. aureus*, *H. haemolyticus*, and *M. catarrhalis*. In contrast, in adults, *Klebsiella pneumoniae*, *Acinetobacter baumannii*, *Pseudomonas aeruginosa*, and *Stenotrophomonas maltophilia* were more prevalent, occupying higher ranks in the pathogen spectrum. Among children, the most frequently identified virus was HRV, followed by herpes viruses [CMV > HHV-7 > EBV > HHV-6 > herpes simplex virus 1 (HSV-1)], respiratory syncytial virus (RSV), human mastadenovirus (HAdV), and human parainfluenza virus (HPIV). For adults, herpes viruses played a predominant role, with EBV and HSV-1 taking higher ranks (EBV > CMV = HSV-1 > HHV-7 > HHV-6). Additionally, *C. albicans*, *P. jirovecii*, and *A. fumigatus* were more frequently detected in adults, whereas atypical pathogens were exclusively detected in children. Statistically significant differences were evident between children and adults in terms of upper respiratory tract infection (*p* = 0.003), chronic obstructive pulmonary disease (COPD, *p* < 0.001), bronchiectasis (*p* < 0.001), diabetes (*p* < 0.001), tumors (*p* = 0.003), sepsis (*p* < 0.001), respiratory failure (*p* < 0.001), severe pneumonia (*p* < 0.001), and hospital stays (*p* < 0.001, Table 2).

**Table 2:** Differences in clinical characteristics between children and adults.

Characteristic	Children (*n* = 149)	Adults (*n* = 60)	*p* value
Main comorbidity, *n* (%)			
Upper respiratory tract infection	44 (29.5)	6 (10.0)	0.003
COPD^a^	0 (0)	8 (13.3)	<0.001
Bronchiectasis	0 (0)	13 (21.7)	<0.001
Diabetes	0 (0)	14 (23.3)	<0.001
Tumor	8 (5.4)	11 (18.3)	0.003
Sepsis	8 (5.4)	28 (46.7)	<0.001
Progression of pulmonary infection			
Respiratory failure	2 (1.3)	38 (63.3)	<0.001
Severe pneumonia	12 (8.1)	27 (45.0)	<0.001
Hospital stays, *n* (%)			<0.001
< 2 weeks	139 (93.3)	23 (38.3)	
≥ 2 weeks	10 (6.7)	37 (61.7)	

^a^COPD, chronic obstructive pulmonary disease.

### 3.4 Clinical impact of tNGS

It is important to highlight that within this study, standard reference results for pathogenic diagnosis were unavailable because of the absence of qualified BALF samples for detection and the lack of quality evaluation for individual sputum samples. Nevertheless, through the application of tNGS, several highly pathogenic and rare/noncolonized microorganisms were identified and deemed clinically relevant within the context of pulmonary infection and final diagnosis. This determination was established through comprehensive reviews based on patient history, symptoms, and imaging findings. Notably, tNGS results directly influenced antibiotic treatment decisions in 21 patients, including 12 patients with *M. pneumoniae* infection, 4 patients with *A. fumigatus*, 3 patients with *M. tuberculosis*, 1 patient with *M. abscessus*, and 1 patient with *C. trachomatis*. Analysis of treatment records for all 209 patients revealed that treatment adjustments were made for 81 patients based on tNGS results, while 128 were not altered. The majority of unadjusted cases were associated with prior empirical medications that effectively covered the detected pathogens. Furthermore, the average experimental turnaround time (TAT) for tNGS was 20.2 h, notably shorter than the 2–5 days required for CMTs. This demonstrated that tNGS offered rapid and stable TAT compared to CMTs, which were substantially affected by presumed pathogens and the detected methods.

## 4 Discussion

The emergence of mNGS has ushered in significant advancements in the realm of infectious disease diagnosis, particularly within the domain of pulmonary infections. mNGS has been embraced by the medical community as a supplementary diagnostic method alongside CMTs, due to its ability to swiftly, accurately, and comprehensively identify pathogens compared to CMTs. However, the considerable cost associated with mNGS has posed a substantial hurdle to its widespread clinical applicability (12–14). This constraint has spurred the development and clinical adoption of tNGS. Notably, tNGS does not merely reduce expenses, but rather, it strikes a balance between cost and detection capabilities. Our previous study indicated that the tNGS technology employed here exhibits comparable performance to mNGS in detecting respiratory pathogens while reducing costs by three-quarters (9), highlighting the potential utility of tNGS in pathogenic diagnosis.

Within this study, we aimed to explore the clinical utility of tNGS for diagnosing pulmonary infections in a patient population unable or unwilling to undergo BALF and to compare its efficacy against CMTs. Accordingly, we employed a tNGS assay targeting 153 pathogens to evaluate its detection performance in sputum samples, addressing concerns related to sample collection, testing costs, TAT, and accessibility. Among the enrolled patients, tNGS identified a greater number of potential pathogens (45 vs. 23), encompassing common or clinically relevant respiratory pathogens, including bacteria, viruses, fungi, and atypical pathogens. Moreover, tNGS exhibited a higher positive detection rate compared to CMTs (96.7% vs. 34.0%). These findings underscore the reality that a considerable portion of patients may harbor potential pathogens that remain undetected despite undergoing CMTs. Furthermore, among the 76 patients exhibiting positive results for both tNGS and CMTs, a substantial 86.8% demonstrated either complete or partial consistency between the two methods. This evidence underscores the promising potential of tNGS in these patients. However, interpreting tNGS results presents significant challenges. A growing body of evidence suggests that the respiratory tract is not a sterile environment and potentially pathogenic microorganisms are ubiquitous (15). The acquisition of noninvasive or minimally invasive sputum samples from the lower respiratory tract is prone to contamination by a patient’s own endogenous upper respiratory tract flora (16, 17). Given that pulmonary infections often arise from the patient’s own flora (18), such contamination can complicate the interpretation of tNGS results in sputum samples. The central question remains: does the presence of potential pathogens indicate colonization or infection? If infection is present, does it affect the upper or lower respiratory tract? However, in most cases, definitively determining whether a specific microorganism has caused infection based solely on sputum sample detection is impractical (19). Ultimately, the conclusive judgment relies on a comprehensive analysis of the patient’s clinical context and the physician’s expertise. In this context, tNGS provides more insights into pathogen information compared to CMTs. Since tNGS can simultaneously detect multiple pathogens at one time, it greatly improve the diagnostic efficiency and assist clinicians to improve the differential diagnosis and identification of mixed infections.

Another primary objective of this study is to investigate the distinctions in the pathogen spectrum between children and adults through the analysis of sputum samples. This endeavor provides valuable insights into the likely potential pathogens or colonizing flora prevalent among patients of varying ages. Bacterial infections exhibited a diverse age-related pattern. Numerous respiratory bacteria establish colonization in the respiratory tract of asymptomatic individuals and can subsequently opportunistically lead to pulmonary infections. This pattern is observed in organisms such as *S. pneumoniae*, *H. influenzae*, and *S. aureus* among children, and *K. pneumoniae*, *A. baumannii*, and *P. aeruginosa* among adults (20–23). A comprehensive 11-year surveillance study of respiratory infectious diseases conducted by the Chinese Center for Disease Control and Prevention validated our findings. This study identified specific age thresholds for the detection rates of various bacteria: 9 years for *S. pneumoniae*, 6 years for *H. influenzae*, 2 years for *S. aureus*, 16 years for *K. pneumoniae*, and 40 years for *P. aeruginosa* (no data available for *A. baumannii*). In relation to DNA viruses, especially herpesviruses detected in this study, distinct age patterns were observed among different types. However, except for CMV, the clinical implications of detecting HSV, EBV, HHV-6, and HHV-7 in the respiratory tract remain unclear, and they rarely lead to pulmonary infections. Existing research suggests that herpesviruses may reactivate in patients with severe infections, malignancies, and transplant recipients, with implications for prognosis and mortality (24–27). In contrast, the age patterns associated with RNA viruses, atypical pathogens, and fungi are comparatively straightforward. We observed that HRV, RSV, HAdV, HPIV, and *M. pneumoniae* were predominantly detected in children, potentially because of their comparatively lower immunity levels than adults and increased opportunities for transmission within school environments (28). Fungal infections were more prevalent in adults, which may be linked to the complex comorbidities and infection severity typically found in this age group. Conditions such as COPD, bronchiectasis, diabetes, malignant tumors, sepsis, and severe pneumonia are all high-risk factors for invasive fungal diseases (29, 30). In alignment with prior research, these outcomes underscore the presence of age-specific heterogeneity in the distribution of respiratory microorganisms. This heterogeneity may play a role in differentiated tNGS interpretation and the identification of relevant pathogens.

While tNGS demonstrated superior detection performance for potential pathogens in sputum samples, our study findings underscore the importance of interpreting positive results with caution. Nonetheless, when integrated with comprehensive clinical analysis, tNGS proves valuable for identifying highly pathogenic and rarely colonizing microorganisms, such as *M. tuberculosis*, certain atypical pathogens, *Aspergillus*, and nontuberculous *Mycobacteria*. Such pathogens are typically less susceptible to colonization. Moreover, treatment adjustments were made in response to tNGS results for 38.8% (81/209) of patients, with tNGS directly guiding antibiotic treatment in 10.0% (21/209) of patients. Patients with unidentified pathogens may benefit from the insights provided by tNGS. Subsequent larger-scale clinical studies are essential to further elucidate the role of tNGS in clinical diagnosis and treatment of pulmonary infections.

This study has several limitations that warrant consideration. First, the absence of standard reference results for pathogenic diagnosis prevented the calculation of sensitivity and specificity for tNGS, thus hindering a comprehensive assessment of its diagnostic performance. Second, distinguishing between microbial colonization and infection posed significant challenges because the current tNGS technology lacks uniform standards for pathogenic diagnosis. Third, the sample size was relatively small, and the study duration was limited, potentially affecting the robustness of the results.

In conclusion, our study underscores the clinical utility of sputum-based tNGS in the pathogenic diagnosis of pulmonary infections among patients who are not candidates for bronchoalveolar lavage. The findings demonstrate that tNGS yields a notably higher positive detection rate compared to CMTs, which aids in the early identification of potential pathogens, particularly those that are highly pathogenic or rarely colonize. This technology also supports clinical treatment decision-making. Additionally, the study reveals age-specific heterogeneity in the distribution of pathogens, indicating the necessity for distinct interpretations of tNGS results among patients of varying ages. Nonetheless, further research is imperative to establish clear indications, criteria for sample selection, and guidelines for result interpretation in the context of tNGS.

## Data availability statement

The original contributions presented in the study are publicly available. This data can be found at: https://www.ncbi.nlm.nih.gov/bioproject/?term=PRJNA1047270.

## Ethics statement

The studies involving humans were approved by Medical Ethics Committee of The First People’s Hospital of Qinzhou. The studies were conducted in accordance with the local legislation and institutional requirements. The human samples used in this study were acquired from primarily isolated as part of your previous study for which ethical approval was obtained. Written informed consent for participation was not required from the participants or the participants’ legal guardians/next of kin in accordance with the national legislation and institutional requirements.

## Author contributions

ZD: Conceptualization, Formal analysis, Software, Visualization, Writing – original draft. CLi: Methodology, Writing – original draft. YiW: Data curation, Validation, Writing – review & editing. FW: Data curation, Validation, Writing – review & editing. CLia: Data curation, Validation, Writing – review & editing. WD: Funding acquisition, Investigation, Resources, Writing – review & editing. YuW: Conceptualization, Funding acquisition, Investigation, Project administration, Supervision, Writing – review & editing.

## Footnotes

^1^https://www.ncbi.nlm.nih.gov
