# Supplementary material for: Targeted next-generation sequencing for pulmonary infection diagnosis in patients unsuitable for bronchoalveolar lavage
Source: Front Med (Lausanne). 2023 Dec 21;10:1321515. doi: 10.3389/fmed.2023.1321515 (PMC10764475; doi:10.3389/fmed.2023.1321515)
Supplement: Supplementary file 3 [file Presentation_1.PDF]

**Supplementary File. Methods of CMTs**

1. Microbial culture

Sputum samples were cultured on blood agar, chocolate agar, and eosin methylene blue agar at 35°C. Sabouraud dextrose agar without chloramphenicol incubated at 35°C was used to isolate fungi. Bacteria were identified by the Vitek2 automated system (BioMérieux, Marcy-l'Etoile, France).

2. Real-time PCR

The artus CMV PCR Kit (4500003, Qiagen, Shanghai, China), artus EBV PCR Kit (4501363, Qiagen, Shanghai, China), and artus HSV-1/2 PCR Kit (4500363, Qiagen, Shanghai, China) for amplification and quantitation were performed in the ABI 7500 Fast and 7500 Real-Time PCR Systems (Thermo Scientific, Massachusetts, USA) according to the manufacturers' instructions.

3. Serologic testing

The indirect immunofluorescent assay (IFA) PNEUMOSLIDE IgM Kit (Vircell, S.L., Granada, Spain) to simultaneously test antibodies against the main ethiological agents causing respiratory tract infectious: *Legionella pneumophila* sg 1, *Mycoplasma pneumoniae*, *Coxiella burnetii*, *Chlamydophila pneumoniae*, adenovirus, respiratory syncytial virus, influenza A, influenza B and parainfluenza 1, 2 and 3.

(1-3)-β-D-Glucan Assay Kit (DNK-1401-1, Dynamiker Biotechnology, Tianjin, China) and Galactomannan Assay Kit (DNK-1402-2, Dynamiker Biotechnology, Tianjin, China) were used for fungal and *aspergillus* detection.
